# A case report of cardiac rehabilitation in coronary artery aneurysm combined with acute ST segment elevation myocardial infarction

**DOI:** 10.1002/ccr3.9507

**Published:** 2024-10-23

**Authors:** Xin‐di Feng, Zi‐lin Ma, Jia‐ying Huang, Jing‐yi Tang, Yi‐hong Wei

**Affiliations:** ^1^ Cardiovascular Department Longhua Hospital Affiliated to Shanghai University of Traditional Chinese Medicine Shanghai China; ^2^ Shanghai University of Traditional Chinese Medicine Shanghai China

**Keywords:** acute ST‐segment elevation myocardial infarction, cardiac rehabilitation, coronary artery aneurysm, heart function

## Abstract

Coronary artery aneurysm (CAA), marked by diffuse or localized dilation of artery, can cause life‐threatening complications in acute coronary syndrome. This report presents a case of CAA combined with acute ST‐segment elevation myocardial infarction, successfully treated with cardiac rehabilitation exercise therapy, offering insights for clinical practice.

## 1 INTRODUCTION

Coronary artery aneurysm (CAA), characterized by the dilation of the coronary arteries beyond the normal diameter, typically ranges from 1.5 to 2 times that of the unaffected vessels.^1^ The prevalence of CAA is relatively low, with an incidence rate estimated to be between 0.2% and 10% among the general population.^2^ Notably, the incidence rate increases to approximately 0.3% to 5% in patients who have undergone percutaneous coronary interventions (PCI).^3^ CAA is more frequently observed in male individuals and tends to affect a single coronary vessel predominantly, with a predilection for the right coronary artery. Subsequently, the left anterior descending and circumflex arteries are also commonly involved, whereas the involvement of the left main is less frequent. Additionally, the occurrence of multi‐vessel involvement in CAA is considered rare.^4^

CAA patients with concurrent acute coronary syndrome (ACS) are noted to have a more adverse prognosis, with increased risks of sudden cardiac death, recurrent myocardial infarction, and stroke.^5^ Furthermore, complications such as PCI failure, stent thrombosis, and distal coronary embolism are also more frequently observed in these patients.^6^ This case report details the management of a patient with CAA who presented with acute ST‐segment elevation myocardial infarction (STEMI). Post‐emergency PCI, the patient was treated with standardized pharmacotherapy complemented by cardiac rehabilitation, resulting in favorable clinical outcomes.

## 2 CASE PRESENTATION

**FIGURE 1:**
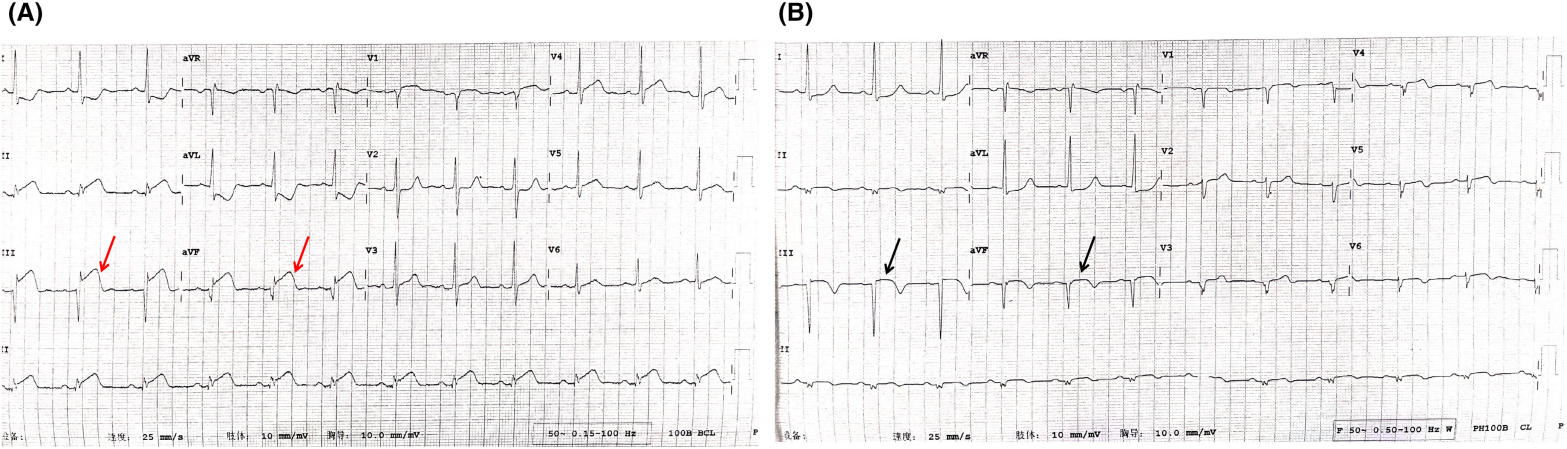
Electrocardiogram before (A) and 1 day after percutaneous coronary intervention (B). Red arrow: Elevated ST segment; Black arrow: Receding ST segment.

A 46‐year‐old male patient presented with a 10‐h history of recurrent precordial chest distress and pain radiating to the right chest, which spontaneously resolved after several tens of minutes. The symptoms recurred at 18:00, accompanied by cold sweats. An electrocardiogram (ECG) at an external facility revealed sinus bradycardia, with 0.1 mV horizontal ST‐segment depression in leads I and aVL, and a convex‐upward ST‐segment elevation in leads II, III, and aVF. Serum level of cardiac troponin I (cTnI) was 0.877 ng/mL. The patient was transferred to our institution for further management by emergency. Upon arrival, ECG was consistent with previous test (see Figure 1A). Additional laboratory investigations demonstrated B‐type natriuretic peptide (BNP) at 25.00 pg/mL, creatine kinase‐MB (CK‐MB) at 9.47 ng/mL, myoglobin (MYO) at 101.8 ng/mL, and a further increase in cTnI to 1.421 ng/mL. The patient subsequently underwent urgent coronary angiography and intervention.

**FIGURE 2:**
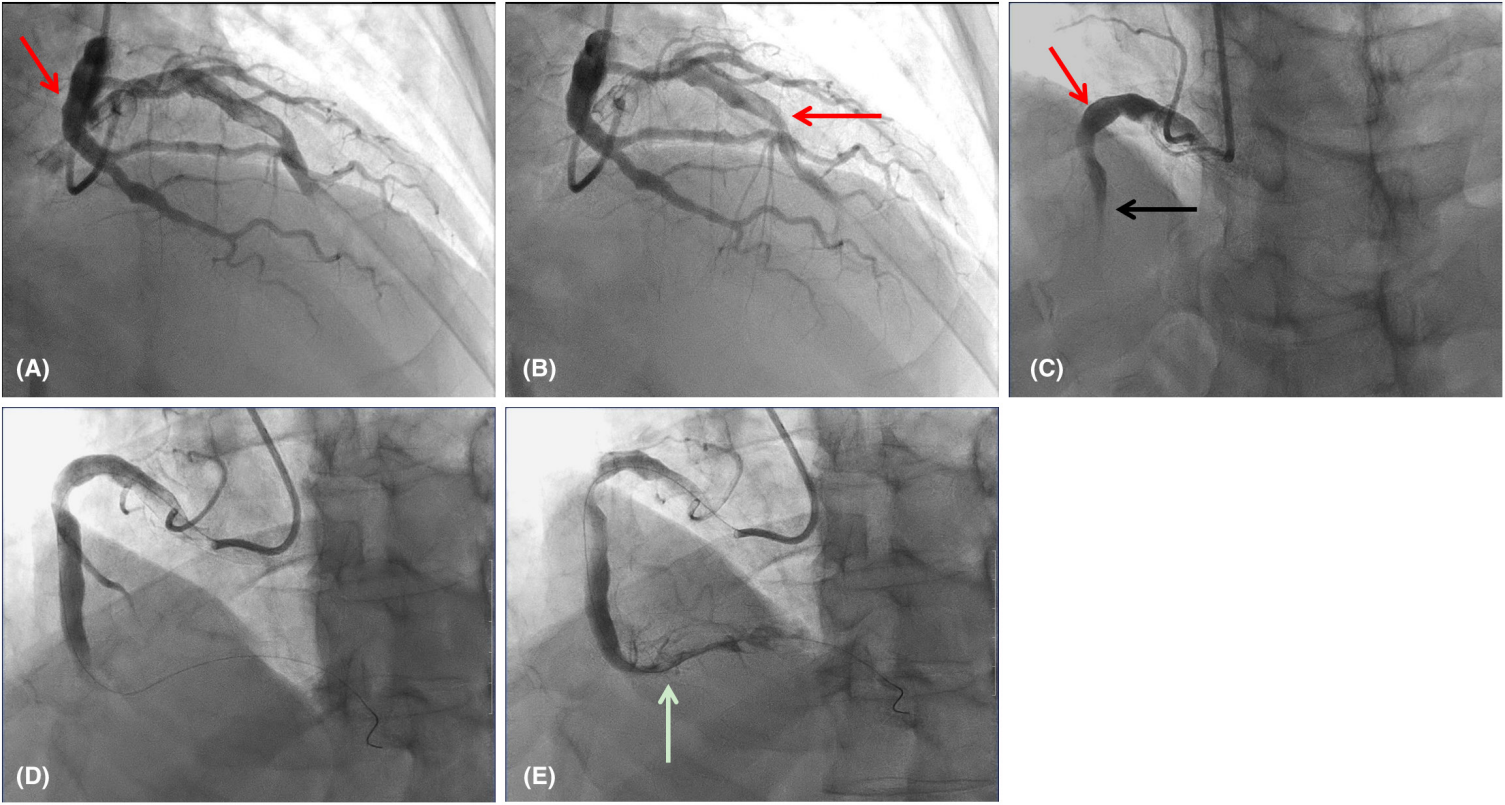
Emergency coronary angiography. (A, B) Coronary artery aneurysm in left anterior descending, circumflex and blunt marginal branches of coronary artery; (C) The right coronary artery exhibits significant dilation with aneurysmal ectasia, and is completely occluded starting from the proximal segment; (D, E) Following percutaneous coronary intervention of the right coronary artery, blood flow has been successfully reestablished. Red arrow: Coronary artery aneurysm; Black arrow: Occluded coronary artery; White arrow: Coronary artery with restored blood flow.

Intraoperative findings included a dilated and irregular left anterior descending artery with aneurysm‐like dilatation, without significant luminal narrowing. The first diagonal branch showed no significant stenosis, but slow distal flow with a TIMI grade of 2 was observed. The left circumflex artery and obtuse marginal branch also exhibited aneurysm‐like dilatation with irregular vessel walls, without significant stenosis, and slow distal flow. The right coronary artery was markedly dilated with aneurysm‐like changes and was completely occluded from its proximal segment (see Figure 2). A large volume of red thrombus was aspirated from the distal right coronary artery, and subsequent angiography demonstrated restoration of antegrade flow with significant thrombus burden in the distal segments. There was a focal 90% stenosis in the mid‐segment of the right coronary artery. Repeated dilations were performed using a cutting balloon and a high‐pressure balloon at the lesion site. Intracoronary administration of tirofiban and recombinant human urokinase were given, with repeated aspiration of large volumes of red thrombus, leading to improved distal flow. The procedure was successful, and the patient was transferred to the cardiac intensive care unit for further management.

The patient was diagnosed with “coronary atherosclerotic heart disease, acute coronary syndrome, acute ST‐segment elevation myocardial infarction (STEMI), Killip class I, CAA, coronary artery thrombosis, and hypertension.”

**FIGURE 3:**
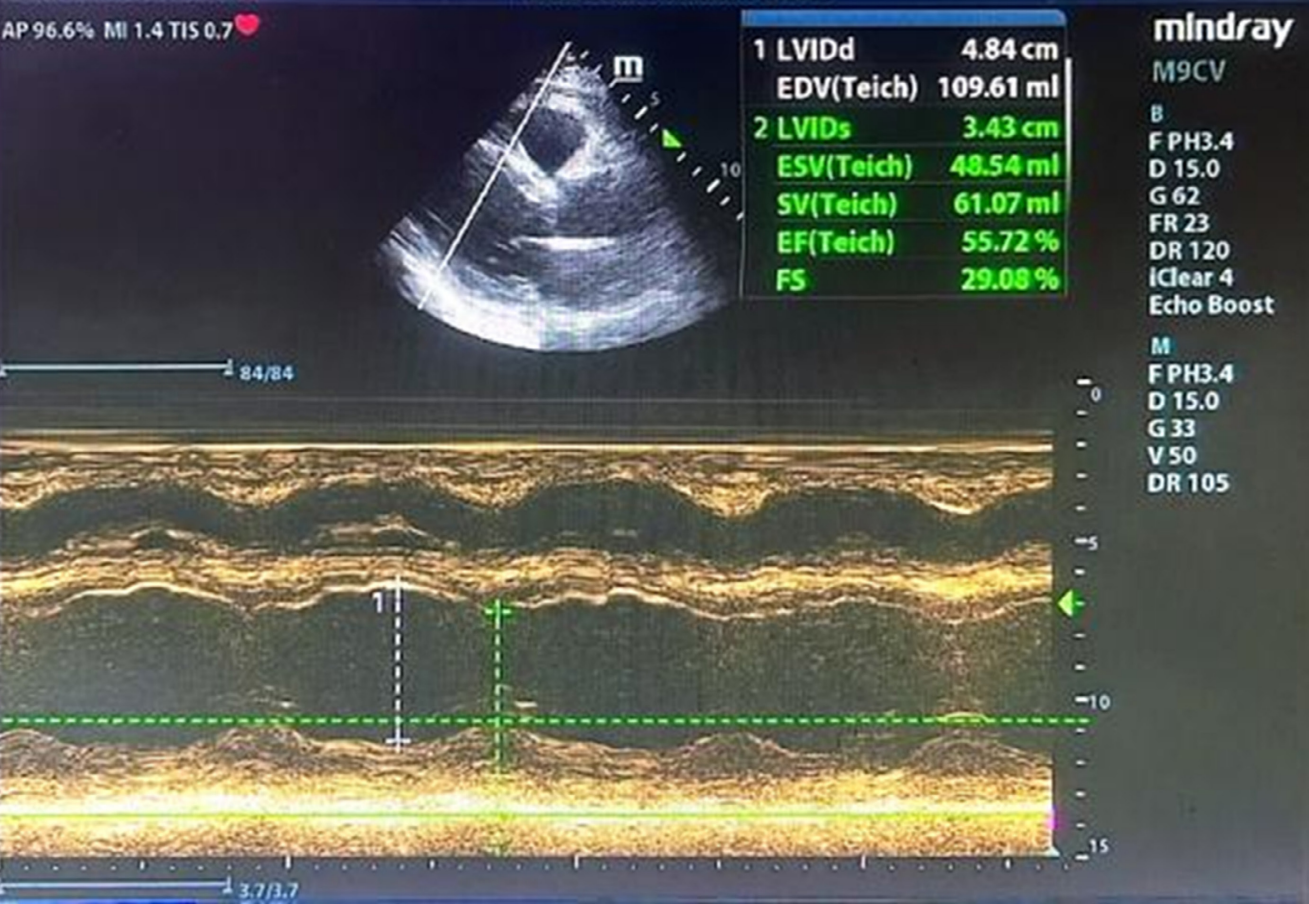
Echocardiography after percutaneous coronary intervention. Left Ventricular Internal Diameter in Diastole (LVIDd): 4.84 cm, End‐Diastolic Volume (EDV): 109.61 mL, Left Ventricular Internal Diameter in Systole (LVIDs): 3.43 cm, End‐Systolic Volume (ESV): 48.54 mL, Left ventricular stroke volume (SV): 61.07 mL, Left ventricular ejection fraction (LVEF): 55.72%, Fractional Shortening (FS): 29.08%.

The patient explicitly denies any past medical history of hypertension, hyperlipidemia, diabetes, cerebrovascular diseases, or other similar conditions. There is no record of infectious diseases, surgical trauma, blood transfusions, or a history of smoking and alcohol consumption. On the second postoperative day, the ST segments of the electrocardiographic leads II, III, and aVF exhibit a pronounced depression, accompanied by the formation of a Q wave. Concurrently, there is an absence of R waves in the precordial leads (see Figure 1B). The biochemical indicators of blood showed: CK‐MB 158.91 ng/mL, MYO 327.1 ng/mL, cTnI 46.000 ng/mL, N‐terminal pro‐brain natriuretic peptide (NT‐proBNP) 265 pg/mL, alanine aminotransferase (ALT) 67 U/L, aspartate aminotransferase (AST) 216 U/L, total bilirubin 26.3 μmol/L, direct bilirubin 5.3 μmol/L, uric acid 544 μmol/L, creatinine 92.4 μmol/L, eGFR‐EPI 85.19%, triglycerides (TG) 1.94 mmol/L, total cholesterol (TC) 4.75 mmol/L, high‐density lipoprotein cholesterol (HDL‐C) 1.01 mmol/L, low‐density lipoprotein cholesterol (LDL‐C) 3.33 mmol/L, very low‐density lipoprotein cholesterol (VLDL‐C) 0.41 mmol/L. Prothrombotic screening, which includes antithrombin III, protein C, protein S, and thyroid function indicators, showed no abnormalities. Echocardiography revealed mild regurgitation of the mitral, tricuspid, and aortic valves, reduced diastolic function of the left ventricle, mild segmental wall motion abnormalities of the myocardium, with a left ventricular ejection fraction (LVEF) of 55% and a left ventricular stroke volume (SV) of 61 mL (see Figure 3). Cardiopulmonary exercise test indicated a mild to moderate decrease in exercise tolerance, primarily due to cardiovascular and muscular limitations, predominantly peripheral skeletal muscle limitations; a lower heart rate (HR) of lactate threshold (HR LT) suggests unsuitable engagement in high‐intensity activities; a lower METs of lactate threshold (METs LT) indicates restricted daily activities. Prognostic risk assessment included a predicted maximum oxygen uptake (VO_2_%Pre) of 52%; a continuous rise in oxygen uptake (VO_2_)/HR; a progressive ΔVO_2_/ΔWatt curve; a flat blood pressure response during exercise; a normal ECG; and exercise cessation due to respiratory distress; the overall risk for the patient is assessed as moderate.

During the hospitalization, the patient was administered a standardized regimen of antiplatelet, anticoagulant, lipid‐lowering and plaque stabilization therapies, along with medications to improve myocardial metabolism and control blood pressure. Following a significant improvement in the patient's condition, he was discharged. Post‐discharge, the patient continued with a combined therapy of medication and cardiac rehabilitation. The exercise rehabilitation program primarily focused on aerobic activities, complemented by flexibility training, resistance training, and core exercises (as detailed in treatment section).

## 3 TREATMENT

During the patient's hospitalization and subsequent post‐discharge period, a comprehensive pharmacological regimen was prescribed, which included: Aspirin (100 mg, once daily), Clopidogrel (75 mg, once daily), Rivaroxaban (15 mg, once daily), Atorvastatin (20 mg, once nightly), Sacubitril/Valsartan (100 mg, twice daily), Amlodipine (5 mg, once daily), Arotinolol (10 mg, twice daily), Trimetazidine (35 mg, twice daily).

The exercise rehabilitation program has been meticulously designed to improve the patient's physical fitness following a scientifically rigorous and structured approach, with an emphasis on aerobic exercises complemented by flexibility training, resistance training, and core training. The specific regimen is as follows:

1. Aerobic Exercise: Engaged in three times per week, each session comprises 45 min (10 min of warm‐up and 35 min on a power bicycle), with an intensity range of 45–65 watts, targeting a HR of 86–88 beats per minute, and a perceived exertion index of 10–13 points based on the Borg scale.^7^
2. Flexibility Training: Lower limb flexibility exercises are performed three times a week, for 30 min each session, while full‐body flexibility training is conducted once a week, for 20 min per session, focusing on enhancing the range of motion and reducing the risk of muscle strains.
3. Resistance Training: Occurs once a week, for 10 min per session, emphasizing lower limb resistance, including isometric training such as wall squats, resistance band exercises for the gluteus medius, and calf raises with concentric training to improve muscle strength and bone density.
4. Core Training: Executed three times a week, for 20 min per session, and includes dynamic and static exercises such as planks, Russian twists, side bridges, and glute bridges. The plank exercise is performed for two sets, each lasting 30 seconds, aiming to strengthen the core muscles that are essential for overall stability and balance.

## 4 OUTCOME AND FOLLOW UP

A coronary angiography conducted 1 month later at an external facility revealed aneurysmal dilatation changes throughout the coronary system; however, no significant stenosis was observed in the left main, anterior descending artery, circumflex artery, or right coronary artery. Echocardiography indicated normal cardiac chamber sizes, mild aortic regurgitation, decreased left ventricular compliance, and normal left ventricular systolic function. Lipid profile results were as follows: TG 2.04 mmol/L, HDL‐C 0.82 mmol/L, and LDL‐C 1.00 mmol/L. Cardiopulmonary exercise test showed a mild decrease in exercise tolerance, mainly limited by peripheral skeletal muscle. The patient's HR LT had improved, and peak oxygen uptake had significantly increased, allowing him to return to a normal level of daily activity. Prognostic risk assessment included a VO_2_%Pre of 77%, a continuous rise in VO_2_/HR, a progressive ΔVO_2_/ΔWatt curve, normal blood pressure response during exercise, a normal ECG, and exercise cessation due to leg soreness, indicating an overall low risk for the patient. It is evident that following the combined medication and cardiac rehabilitation therapy, there was an overall improvement in the patient's functional capacity (see Table 1).

**TABLE 1:** Cardiopulmonary exercise test before and 1 month after cardiac rehabilitation.

	Before cardiac rehabilitation	1 month after cardiac rehabilitation	Reference value
VO_2_LT, ml	840	890	940
VO_2_peak, ml	1210	1820	2390
VO_2_%Pre, %	52	77	85
VO_2_/kg LT, ml/kg	11.1	11.7	12.4
VOR/kg peak, ml/kg	16.0	23.9	31.0
METs LT, Mets	3.2	3.3	3.7
METs peak, Mets	4.6	6.8	10.9
HR LT, bpm	88	96	
Power MAX, w	96	120	

Abbreviations: HR, heart rate; LT, lactate threshold; METs, metabolic equivalents; VO_2_, oxygen uptake, VO_2_%Pre, predicted maximum oxygen uptake, VOR, volume of oxygen.

## 5 DISCUSSION

CAA is etiologically diverse, with atherosclerosis being a predominant cause. Atherosclerotic processes can diminish vascular wall elasticity, consequently reducing the coronary artery's resilience to intraluminal pressure, thereby predisposing to dilation and aneurysm formation.^8^ Beyond atherosclerosis, inflammatory conditions such as Kawasaki disease, Marfan syndrome, and lupus, as well as non‐inflammatory factors including interventions, toxins, and congenital issues, have been implicated in the genesis of CAA. Despite these associations, the precise pathophysiological mechanisms underlying CAA remain to be fully elucidated and are suspected to interplay with atherosclerotic, inflammatory, and genetic factors.^9^

Diagnostic approaches to CAA are multimodal, encompassing coronary angiography (CAG), intravascular ultrasound (IVUS), optical coherence tomography (OCT), and computed tomography angiography (CTA). CAG affords an assessment of aneurysm diameter and morphology, alongside hemodynamic abnormalities, yet it falls short in detailing vascular structural anomalies, plaque remodeling, and the specifics of aneurysm orifices.^10^ IVUS is recognized for its sensitivity in detecting nascent coronary changes, establishing it as a diagnostic gold standard for CAA. In contrast, OCT provides granular insights into the intimal layer and pathologic conditions.^11^ CTA, with its capability to delineate coronary calcifications, luminal dimensions, thrombi, and aneurysm characteristics, emerges as a pivotal modality for the longitudinal surveillance of CAA progression.^12^, ^13^

In the case presented, the patient, diagnosed with ACS, exhibited pronounced CAA upon CAG, obviating the need for further investigation with IVUS or OCT. The management of CAA is multifaceted, encompassing pharmacological interventions, interventional therapies, and surgical approaches, each demonstrating clinical efficacy; however, there is a notable absence of large‐scale clinical trials to substantiate these treatment modalities.^10^ Antiplatelet agents are instrumental in pharmacological strategies, with a study by Yasara et al. revealing significantly heightened plasma levels of p‐selectin, β‐thromboglobulin, and platelet factor 4 in patients with isolated CAA, underscoring the pronounced platelet activity in these patients.^14^ Consequently, antiplatelet medications, particularly aspirin, constitute the foundation of CAA therapeutics.^9^ Nevertheless, the role of antithrombotic therapy, dual antiplatelet therapy, and the prophylaxis of complications in CAA necessitate further investigation and appraisal. In the clinical case presented, the patient exhibited substantial thrombus during PCI, prompting an enhanced postoperative antithrombotic regimen that included dual antiplatelet therapy with aspirin and clopidogrel, as well as anticoagulation with rivaroxaban. A subsequent one‐month follow‐up revealed no symptoms of chest distress or pain, and coronary angiography demonstrated the absence of thrombus, stenosis, or occlusion, indicative of a satisfactory therapeutic outcome. However, the utility of combined antiplatelet and anticoagulant therapy in CAA patients remains an open question that merits additional research and deliberation.

Cardiac rehabilitation is a multidisciplinary approach that offers comprehensive and systematic prevention for cardiovascular diseases at various stages. It is recognized as a Level I recommendation for the prevention and treatment of cardiovascular diseases in developed countries such as the United States and Europe.^15^ Cardiac rehabilitation exercises not only effectively intervene in risk factors for cardiovascular diseases, such as hypertension, hyperlipidemia, and hyperglycemia, but also enhance patients' exercise capacity, decelerate the progression of coronary atherosclerosis, reverse plaque formation, prevent vascular stenosis or thrombosis, and concurrently improve cardiac function and reduce NT‐proBNP levels.^16^ Cardiac rehabilitation significantly ameliorates the physiological and quality‐of‐life outcomes for patients post‐myocardial infarction, as evidenced by enhancements in the 6‐minute walk test and maximal oxygen uptake.^17^ It also exerts salutary effects on smoking cessation, dietary habits, and weight management, which are pivotal for elevating the quality of life and ensuring long‐term health.^18^ A retrospective analysis of 405 patients with acute myocardial infarction revealed that cardiac rehabilitation notably improves cardiac diastolic function.^19^ In another retrospective study encompassing 174 myocardial infarction patients with metabolic syndrome, high‐intensity interval training was observed to alleviate symptoms and ameliorate body fat composition.^20^ A prospective cohort study conducted in Germany, integrating data from 20 cardiac rehabilitation centers and involving 1408 myocardial infarction patients, demonstrated that cardiac rehabilitation effectively reduces LDL‐C levels and enhances cardiac function, enabling the majority of patients to resume their full‐time employment.^21^ And in the geriatric population, a multicenter, randomized controlled clinical trial focused on patients over 70 years old with ACS indicated that an early, individualized, and cost‐effective exercise intervention can enhance functional capacity and quality of life.^22^ These findings underscore the positive influence of cardiac rehabilitation in post‐myocardial infarction. However, the impact of such rehabilitation on CAA remains unclear, necessitating further investigation. This case, as an instance of a patient with CAA complicated by STEMI, exemplifies the severity and particularities of the condition. Following standardized pharmacological treatment comprising antiplatelet, anticoagulant, and myocardial nourishment therapies, the initiation of cardiac rehabilitation exercises postoperatively facilitated the recovery of various physiological functions, yielding tangible clinical outcomes with reference significance.

## AUTHOR CONTRIBUTIONS

**Xin‐di Feng:** Data curation; methodology; validation; writing – original draft. **Zi‐lin Ma:** Data curation; methodology; validation; writing – original draft. **Jia‐ying Huang:** Data curation; methodology; validation; writing – original draft. **Jing‐yi Tang:** Conceptualization; data curation; formal analysis; investigation; methodology; writing – review and editing. **Yi‐hong Wei:** Conceptualization; data curation; formal analysis; investigation; methodology; writing – review and editing.

## FUNDING INFORMATION

No funding.

## CONFLICT OF INTEREST STATEMENT

The authors have no conflicts of interest to declare.

## ETHICS STATEMENT

Written informed consent was obtained from the patient to publish this report in accordance with the journal's patient consent policy.

## CONSENT

Written informed consent was obtained from the patient to publish this report in accordance with the journal's patient consent policy.

## Data Availability

The data that support the findings of this case study are available within the manuscript.
